# Breast cancer: A review of risk factors and diagnosis

**DOI:** 10.1097/MD.0000000000036905

**Published:** 2024-01-19

**Authors:** Emmanuel Ifeanyi Obeagu, Getrude Uzoma Obeagu

**Affiliations:** a Department of Medical Laboratory Science, Kampala International University, Kampala, Uganda; b School of Nursing Science, Kampala International University, Kampala, Uganda.

**Keywords:** breast cancer, cancers, diagnosis, mortality, prevention, risk factors

## Abstract

Breast cancer remains a complex and prevalent health concern affecting millions of individuals worldwide. This review paper presents a comprehensive analysis of the multifaceted landscape of breast cancer, elucidating the diverse spectrum of risk factors contributing to its occurrence and exploring advancements in diagnostic methodologies. Through an extensive examination of current literature, various risk factors have been identified, encompassing genetic predispositions such as BRCA mutations, hormonal influences, lifestyle factors, and reproductive patterns. Age, family history, and environmental factors further contribute to the intricate tapestry of breast cancer etiology. Moreover, this review delineates the pivotal role of diagnostic tools in the early detection and management of breast cancer. Mammography, the cornerstone of breast cancer screening, is augmented by emerging technologies like magnetic resonance imaging and molecular testing, enabling improved sensitivity and specificity in diagnosing breast malignancies. Despite these advancements, challenges persist in ensuring widespread accessibility to screening programs, particularly in resource-limited settings. In conclusion, this review underscores the importance of understanding diverse risk factors in the development of breast cancer and emphasizes the critical role of evolving diagnostic modalities in enhancing early detection. The synthesis of current knowledge in this review aims to contribute to a deeper comprehension of breast cancer’s multifactorial nature and inform future directions in research, screening strategies, and preventive interventions.

## 1. Introduction

One of the frequent and numerous malignant tumors that affect women is breast cancer. Breast cancer develops and occurs as a result of several internal and external factors.^[1–3]^ Poor lifestyle choices, environmental factors, and social-psychological factors are all linked to its occurrence. It has been demonstrated that 5% to 10% of breast cancers can be attributed to genetic mutations and family history, and 20% to 30% of breast cancers can be attributed to factors that may be modifiable.^[4]^ Breast cells are where breast cancer first develops. A collection of cancer cells known as a cancerous tumor is capable of spreading into and destroying nearby tissue. As well as spreading throughout the body, it can. Breast cells occasionally undergo changes that prevent them from growing or behaving normally. Non-cancerous breast conditions atypical hyperplasia and cysts may result from these changes. Additionally, they may result in benign tumors like intraductal papillomas.^[5]^

However, breast cancer can occasionally result from changes to breast cells. Breast cancer typically begins in the cells that line the ducts, which are the tubes that carry milk from the glands to the nipple. Ductal carcinoma is the name given to this subtype of breast cancer. The cells of the lobules, which are the collections of milk-producing glands, can also give rise to cancer.^[6,7]^ Lobular carcinoma is the name of this type of cancer. Both ductal and lobular carcinomas can be in situ, which means the cancer is still present in the area where it first appeared and has not spread to adjacent tissues. They may also be invasive, which indicates that they have spread into the tissues around them.^[8]^ Breast cancer can also manifest itself in less common forms. These include triple-negative breast cancer, breast Paget disease, and inflammatory breast cancer. Non-Hodgkin lymphoma and soft tissue sarcoma are uncommon forms of breast cancer.^[9]^ Despite having a low incidence of breast cancer, studies show that it has been steadily rising in China. By 2022, the number of Chinese women who will have the disease will surpass 100 per 100,000, and there will be 2.5 million women with the disease overall, aged 35 to 49. Thus, it is crucial to research breast cancer risk factors to lower the disease’s incidence.^[10]^ Breast cancer is the most prevalent cancer in women worldwide and the main reason why women die from cancer. About 630,000 women lost their lives to breast cancer in 2018, and there were approximately 2.09 million newly diagnosed cases. While there are regional variations in breast cancer incidence, it is rising. Due to China’s large population and high incidence of breast cancer, which ranks first globally and has increased over the past few years (17.6% and 15.6%, respectively), even though the incidence of breast cancer (36.1/105) and mortality (8.8/105) are both relatively low worldwide. The burden of the disease is rising alongside the incidence of breast cancer globally, which has grown to be a significant issue for global public health.^[11]^ Breast cancer is a multifactorial disease with major genetic, environmental, and behavioral/lifestyle components. The objective of the current review was to investigate the epidemiology and associated risk factors of breast cancer globally to comprehend its prevalence and aid in early detection. The main risk factors for breast cancer are genetic factors, specifically family history; diet, and obesity, as the quality of life in our country improves, women are getting more and more obese, and their diet tends to be more and more high-fat; smoking and drinking; the other is ionizing radiation; still have, specifically menstruation, bear, and whether lactation, these factors also can affect the occurrence of breast cancer. To lessen the impact exogenous hormones, have on the body, we should try to avoid using cosmetics that contain estrogen in our daily lives. Around these appeals, there has been a lot of debate. As a result, it is essential to thoroughly examine the risk factors for breast cancer using meta-methods to direct clinical prevention and treatment.^[12]^ We conducted a meta-analysis of breast cancer risk factors in Chinese women in the current study by gathering pertinent literature from 2001 to 2021, even though Chinese scholars have already done so.^[13]^ Our goal was to provide fundamental information for the prevention of breast cancer in Chinese women. Something that raises your chance of getting cancer is a risk factor. A habit, substance, or illness could be the culprit. Numerous risk factors contribute to the majority of cancers. However, breast cancer can occasionally develop in women who don’t have any of the risk factors listed below. Women are more likely than men to develop breast cancer. Women are more likely to develop breast cancer when estrogen and progesterone are exposed to their breast cells.

Some breast cancers are aided in their growth by these hormones, particularly estrogen, which has been linked to breast cancer. Canada, the United States, and a few European nations are examples of high-income, developed nations where breast cancer is more prevalent. Age raises the likelihood of getting breast cancer. Women between the ages of 50 and 69 are the most common demographic for breast cancer.^[14]^ The most frequently diagnosed cancer in women and a major global health concern is breast cancer. Researchers have found several risk factors that can raise a woman’s likelihood of getting breast cancer, even though the disease’s precise cause is still unknown. For early detection, prevention, and efficient management of breast cancer,^[14]^ it is essential to comprehend these risk factors. With more than 1 in 10 new cancer diagnoses each year, breast cancer is the most common cancer in women. In the entire world, it is the second most typical cause of cancer-related death in females. Milk-producing glands are located in front of the chest wall on the anatomy of the breast. They rest on the pectoralis major muscle, and the breast is supported by ligaments that join it to the chest wall. The breast is made up of 15 to 20 lobes that are arranged in a circle. The size and shape of the breasts are determined by the fat that covers the lobes. Each lobe is made up of lobules that contain the glands that produce milk when hormones are stimulated. Breast cancer always progresses subtly. The majority of patients learn they have their illness while getting their regular screenings. Others may exhibit nipple discharge, a breast shape or size change, or an unintentionally discovered breast lump. Mastalgia is not unusual, though. Breast cancer diagnosis requires a physical examination, imaging, particularly mammography, and tissue biopsy.^[14]^

Early diagnosis increases the likelihood of survival. Poor prognosis and distant metastasis are caused by the tumor’s propensity to spread lymphatically and hemologically. This clarifies and highlights the significance of programs for breast cancer screening.^[15]^ Anything that raises the possibility of developing cancer is a risk factor. It might be a habit, substance, or illness. Many risk factors combine to cause the majority of cancers. Women are more likely than men to develop breast cancer. Women are more likely to develop breast cancer when estrogen and progesterone are exposed to their breast cells. Breast cancer is more prevalent in high-income, developed nations like Canada, the United States, and some European nations. These hormones, particularly estrogen, are linked to the disease and promote its growth. As you get older, your risk of developing breast cancer rises. Most breast cancer cases in women are diagnosed between the ages of 50 and 69 years.^[14]^

## 2. Risk factors for developing breast cancer among women

### 2.1. Personal history of breast cancer

An increased risk of breast cancer recurrence exists in women who have previously experienced it. The second breast cancer may appear in the same breast as the first one or in a different breast. Although the majority of women who have ductal carcinoma in situ or lobular carcinoma in situ breast cancers do not recur, these women are at an increased risk of doing so.^[16]^

### 2.2. Breast and other types of cancer in the family history

The presence of breast cancer in one or more close blood relatives indicates that the disease runs in the family. More breast cancer cases than one might anticipate randomly occur in some families. It can be difficult to determine whether a family’s history of cancer is the result of coincidence, a common lifestyle, genes passed down from parents to children, or a combination of these factors.^[17]^

### 2.3. Mutations in the BRCA gene

An altered gene is referred to as a genetic mutation. Certain types of cancer may be more likely to develop as a result of some gene changes. A parent can pass on inherited gene mutations to their offspring. Only a small percentage of breast cancers (roughly 5%–10%) are brought on by inherited gene mutations. Normal human physiology includes both BRCA1 and BRCA2, which are breast cancer genes. As a result of what seems to be their involvement in regulating the growth of cancer cells, these genes are known as tumor suppressors. BRCA1 or BRCA2 gene mutations may cause them to lose their ability to regulate the development of cancer. Rarely occur these mutations. Roughly 1 in 500 people experience them. A mutated BRCA gene can be inherited by both men and women from either their mother or father. Children of those who carry the gene mutation may also inherit it. A child has a 50% chance of inheriting the gene mutation if 1 of the 2 copies of the *BRCA* gene has the mutation in 1 or both parents. A child also has a 50% chance of not inheriting the gene mutation, according to this.^[18]^ According to studies, women who inherit *BRCA1* or *BRCA2* gene mutations have an 85% lifetime risk of developing breast cancer. Additionally, compared to other women, those who carry these inherited mutations are at an increased risk of developing breast cancer earlier in life. Breast cancer in both breasts is more likely to strike women who have the BRCA gene mutation. They are more likely to get cancer in the other breast if they have cancer in 1 breast. Ovarian cancer can strike a woman at any age if she carries a BRCA gene mutation.^[19]^

### 2.4. Large breasts

Compared to fatty tissue, dense breasts have more milk ducts, glands, and connective tissue. Breast density is a genetic trait. Compared to women with little or no dense breast tissue, women with dense breast tissue have a higher risk of developing breast cancer. Breast density can only be detected by a mammogram, but dense breasts also make the image more difficult to interpret. On a mammogram, dense tissue appears white, like tumors, while fatty tissue appears dark, concealing a tumor.^[20]^

### 2.5. The late menopause

The body’s level of hormones, primarily estrogen and progesterone, begins to decline as the ovaries stop producing them, resulting in menopause. A woman’s menstrual cycle is stopped as a result of this. Your cells are exposed to estrogen and other hormones for a longer period if you enter menopause later in life (after age 55). This raises the possibility of breast cancer. Likewise, breast tissue is exposed to estrogen and other hormones for a shorter period when menopause occurs earlier in life. A lower risk of breast cancer is associated with early menopause.^[21]^

### 2.6. Whether there are late or no pregnancies

Breast cells’ exposure to circulating estrogen is halted during pregnancy. It also reduces the overall number of menstrual cycles a woman experiences throughout her lifetime. A woman’s risk of breast cancer is marginally higher than it is for a woman who has at least one full-term pregnancy before the age of 30. Reduced risk of breast cancer is associated with early pregnancy. A woman is more protected from breast cancer the more children she has. Breast cancer risk is increased if a woman never conceives.^[22]^

### 2.7. Hormonal replacement treatment

According to the Women’s Health Initiative (WHI) study, estrogen alone increased breast cancer risk by about 1% per year and combined hormone replacement therapy (HRT) increased risk by about 8% per year. The study also discovered that, in comparison to a placebo, the risk increased even with relatively brief use of combined HRT. After stopping HRT for a few years, the higher risk seems to be gone. The WHI study also revealed that, among Canadian women aged 50 to 69, there was a notable decline in the number of new cases of breast cancer between 2002 and 2004. The use of combined HRT decreased at the same time as this drop. Other nations around the world, such as the United States, Australia, Germany, the Netherlands, Switzerland, and Norway, have also noticed this trend. The risks associated with the long-term use of combined HRT are now thought to outweigh the advantages.^[23]^

### 2.8. Being overweight

In post-menopausal women, obesity increases the risk of developing breast cancer. According to studies, women with a body mass index of 31.1 or higher who have never used HRT are 2.5 times more likely to develop breast cancer than those with a body mass index of 22.6 or lower. In particular, estrogens from the ovaries play a significant role in breast cancer. Many breast cancer risk factors are thought to be caused by the cumulative estrogen dose that the breast tissue absorbs over time. The majority of the body’s estrogen is produced by the ovaries, but after menopause, fat tissue only produces a small amount of estrogen. A higher estrogen level can result from having more fat tissue, which raises the risk of breast cancer.^[24]^

### 2.9. Estrogen

Breast cancer risk is linked to estrogens, both endogenous and exogenous. In premenopausal women, the ovary typically produces endogenous estrogen, and ovarian removal can lower the risk of breast cancer. HRT and oral contraceptives are the main exogenous estrogen sources. Since the 1960s, oral contraceptives have been extensively used, and their formulations have been improved to minimize side effects. The odd ratio is still higher than 1.5 for Iranian and African American female populations, though. Oral contraceptives do not, however, raise the risk of breast cancer in women who stop using them for more than 10 years. For menopausal or postmenopausal women, HRT entails the administration of exogenous estrogen or other hormones. The use of HRT can raise the risk of breast cancer, according to several studies. According to the Million Women Study in the UK, there is a 1.66 relative risk between those who currently use HRT and those who have never used it. A cohort study of 22,929 Asian women found that after using HRT for 4 and 8 years, respectively, hazard ratios (HRs) of 1.48 and 1.95 were found. After 2 years of stopping HRT, it has been demonstrated that the risk of breast cancer significantly declines. With a 3.6 HR for a new breast tumor, the recurrence rate is also high among breast cancer survivors who take HRT. Since the negative effects of HRT were revealed in 2003 based on the WHI randomized controlled trial, there has been a 7% decrease in the incidence rate of breast cancer in America.^[25]^

## 3. Breast cancer in women: Diagnosed through appended technologies

Breast tumors typically start as benign tumors or even metastatic carcinomas due to ductal hyperproliferation, which is then constantly stimulated by various carcinogenic factors. Breast cancer is initiated and progresses differently depending on the microenvironment of the tumor, such as stromal influences or macrophages. When only the stroma of the rat mammary gland was exposed to carcinogens—not the extracellular matrix or the epithelium—neoplasms could be induced. A mutagenic inflammatory microenvironment that macrophages can create can encourage angiogenesis and help cancer cells avoid immune rejection. Different DNA methylation patterns between the typical and tumor-associated microenvironments have been observed, suggesting that epigenetic changes in the tumor microenvironment can encourage carcinogenesis. Cancer stem cells (CSCs), a new subclass of malignant cells within tumors, have recently been identified and linked to tumor initiation, escape, and recurrence. This small population of cells, which may originate from stem cells or progenitor cells in healthy tissues, can regenerate itself and is resistant to traditional treatments like chemotherapy and radiotherapy. Ai Hajj was the first to identify breast cancer stem cells (bCSCs), and immunocompromised mice could develop new tumors from as few as 100 bCSCs. As opposed to basal stem cells, luminal epithelial progenitors are more likely to be the source of bCSCs. The self-renewal, proliferation, and invasion of bCSCs are mediated by signaling pathways that include Wnt, Notch, Hedgehog, p53, PI3K, and HIF. More research is nevertheless required to comprehend bCSCs and create fresh methods for their complete eradication.^[19]^

The CSC theory and the stochastic theory are 2 speculative theories for how breast cancer starts and spreads. According to the theory about CSCs, all subtypes of tumors are descended from the same stem cells or transit-amplifying cells. A variety of tumor phenotypes are caused by acquired genetic and epigenetic mutations in stem cells or progenitor cells. According to the stochastic theory, a single type of cell is the source of all tumor subtypes. Any breast cell can gradually develop random mutations, and when enough mutations have accumulated, the breast cell can transform into a tumor cell. Even though both theories have a lot of data to back them up, neither can fully explain how human breast cancer first developed.^[26]^

## 4. Biology-based breast cancer prevention

To enhance the quality of life for breast cancer patients, biological prevention, primarily known as monoclonal antibodies for the disease, has recently been developed. These monoclonal antibodies have human epidermal growth factor receptor 2 (HER2) as one of their primary targets. The HER2 protein is overexpressed or the HER2 gene is amplified in about 20% to 30% of all breast cancer cases. The first HER2-targeted medication to receive FDA approval is trastuzumab (Herceptin), a recombinant humanized monoclonal antibody. It can directly interact with the C-terminal region of domain IV in the extracellular region of HER2. Trastuzumab’s anti-tumor mechanism has not yet been fully understood. Trastuzumab may inhibit the growth and proliferation of cancer cells through several possible mechanisms, including activating the immune system against cancer cells through an effect known as antibody-dependent cell-mediated cytotoxicity, inhibiting the MAPK and PI3K/Akt pathways, and enlisting ubiquitin to internalize and degrade HER2. With an objective response rate of 26%, trastuzumab was initially used to treat metastatic breast cancer. Trastuzumab interacts favorably with other anti-tumor medications, including nimotuzumab, carboplatin, 4-hydroxycyclophosphamide, docetaxel, and vinorelbine, according to in vitro studies. According to the HERA and TRAIN trials, chemotherapy given in combination with adjuvant trastuzumab for a year can prolong disease-free survival in HER2+ breast cancer patients (HR = 0.76). Trastuzumab plus docetaxel was shown to be more effective than docetaxel alone in treating HER2-positive metastatic breast cancer, with an objective response rate of 50% versus 32%, in a randomized phase II trial carried out by Marty. Patients receiving trastuzumab, however, also experienced adverse effects like congestive heart failure and a decline in their left ventricular ejection fraction.^[4]^

## 5. Breast cancer in women is diagnosed

### 5.1. Mammography

Diagnostic mammography is an x-ray that creates an image of the breast using low radiation doses. It is used to follow up on unexpected findings from a clinical breast exam or a screening mammogram. It is also possible to use mammography during a biopsy to identify an abnormal area.^[27]^

### 5.2. Ultrasound

An ultrasound creates images of various body parts using high-frequency sound waves. It is used to determine whether a lump in the breast is a solid tumor or a cyst. Additionally, ultrasound can be used by medical professionals to direct them to the biopsy site. An ultrasound may be performed on women with advanced breast cancer to determine whether liver metastasis has occurred.^[28]^

### 5.3. Biopsy

Breast cancer can only be accurately identified through a biopsy. The purpose of a biopsy is to remove tissues or cells from the patient’s body for laboratory testing. The pathologist’s report will determine whether or not cancer cells were discovered in the sample. The type of biopsy performed will depend on whether the lump is palpable, meaning you can feel it, or non-palpable, meaning you can’t. To locate the area to be tested, the doctor may use ultrasound or mammography. The majority of biopsies are performed in a hospital, and once they are complete, you can leave for home.^[29]^

### 5.4. The core biopsy

Removes tissue from the body using a unique hollow needle. It is employed by doctors to obtain a sample from a breast region that is thought to be suspicious. During the procedure, they might take several samples from the area. To remove more tissue through the hollow needle, doctors occasionally use a special vacuum. Vacuum-assisted core biopsy is the name of this method.^[17]^

### 5.5. A lymph node biopsy

A lymph node biopsy is a surgical procedure that involves the removal of lymph nodes so they can be examined under a microscope to determine if they contain cancer. Breast cancer cells can separate from the tumor and move through the lymphatic system. Lymph nodes beneath the arm are where they might spread first. To help determine the stage of breast cancer, doctors count the number of lymph nodes that contain the disease.^[30]^

### 5.6. Fine needle aspiration

Removes a small amount of tissue from a lump using a syringe and a very thin needle. It helps doctors determine whether a lump is a cyst or a solid tumor. Whether a cancer is non-invasive or invasive cannot be determined by fine needle aspiration (FNA).^[31]^ During the procedure, a healthcare professional inserts a thin needle into the breast lump, guided by palpation or imaging techniques such as ultrasound. A syringe attached to the needle is used to suction out cells or fluid from the lump. These cells or fluid samples are then examined under a microscope by a pathologist to determine if they are cancerous (malignant) or noncancerous (benign). FNA is a minimally invasive procedure that can provide valuable information about the nature of the breast lump. It helps in the diagnosis of breast cancer by analyzing the characteristics of the cells, aiding in determining the presence of cancerous cells, and guiding further diagnostic or treatment procedures. However, depending on the situation, additional tests like a core needle biopsy or surgical biopsy may be recommended for a more comprehensive evaluation.

## 6. Preventative measures and ongoing research efforts

Certainly, preventive measures and ongoing research efforts are crucial components in the fight against breast cancer.

## 7. Preventative measures

Encouraging women to undergo regular mammograms and screenings based on age and risk factors can aid in early detection, leading to better treatment outcomes.^[32]^ Promoting a healthy lifestyle that includes maintaining a balanced diet, regular exercise, limiting alcohol consumption, avoiding smoking, and maintaining a healthy weight can reduce the risk of developing breast cancer. Encouraging breastfeeding, which has been shown to have protective effects against breast cancer, can be promoted as a preventive measure.^[33]^ Providing comprehensive and accessible education on breast cancer risks, symptoms, and the importance of early detection can empower individuals to take proactive measures and seek timely medical attention. For individuals with a family history or known genetic mutations (like BRCA1 or BRCA2), genetic counseling and testing can help in assessing risks and making informed decisions about preventive measures.^[34]^ Understanding the risks associated with certain hormone therapies and discussing alternatives with healthcare providers, particularly for menopausal symptoms, is important.

## 8. Ongoing research efforts

Research continues to develop targeted therapies that focus on specific genetic mutations or molecular markers associated with breast cancer, improving treatment efficacy while reducing side effects.^[35]^ Investigating the role of immunotherapy in breast cancer treatment, harnessing the body’s immune system to target cancer cells, is an area of active research. Ongoing research aims to develop more sensitive and specific screening methods beyond mammography, including molecular imaging and blood-based biomarkers, for earlier and more accurate diagnosis. Studying genetic and epigenetic factors influencing breast cancer development helps in identifying new targets for therapy and understanding individual susceptibility.^[36]^ Research focuses on identifying additional lifestyle modifications, medications, or interventions that can further reduce the risk of developing breast cancer. Collaborative research initiatives between countries and institutions aim to share data, resources, and expertise, advancing our understanding of breast cancer and improving treatment outcomes globally. By continuing to prioritize prevention through lifestyle modifications, early detection through effective screening, and investing in cutting-edge research, the hope is to reduce the incidence, morbidity, and mortality associated with breast cancer worldwide.

## 9. Conclusion

Breast cancer remains a significant global health concern, impacting millions of individuals each year. This review has underscored the multifaceted nature of breast cancer, highlighting various risk factors and diagnostic approaches crucial in understanding and managing this disease. Moreover, advancements in diagnostic techniques have significantly improved early detection and treatment outcomes. Mammography, alongside emerging technologies like magnetic resonance imaging and molecular testing, plays a pivotal role in identifying breast cancer at its early stages, enabling prompt intervention and potentially improving patient prognoses. Moving forward, continued research into identifying additional risk factors, enhancing screening methods, and developing targeted therapies remains imperative. Furthermore, promoting awareness, advocating for increased screening accessibility, and fostering global collaboration among medical professionals and researchers is crucial in the ongoing fight against breast cancer. A comprehensive approach that integrates research, education, early detection, and accessible healthcare services is essential in combating breast cancer and reducing its impact on individuals and societies worldwide.

## Author contributions

**Conceptualization:** Emmanuel Ifeanyi Obeagu, Getrude Uzoma Obeagu.

**Methodology:** Emmanuel Ifeanyi Obeagu.

**Resources:** Emmanuel Ifeanyi Obeagu, Getrude Uzoma Obeagu.

**Supervision:** Emmanuel Ifeanyi Obeagu, Getrude Uzoma Obeagu.

**Visualization:** Emmanuel Ifeanyi Obeagu, Getrude Uzoma Obeagu.

**Writing – original draft:** Emmanuel Ifeanyi Obeagu, Getrude Uzoma Obeagu.

**Writing – review & editing:** Emmanuel Ifeanyi Obeagu, Getrude Uzoma Obeagu.

**Validation:** Getrude Uzoma Obeagu.
